# Editorial: Uncovering a multidisciplinary approach in pediatric dentistry

**DOI:** 10.3389/fdmed.2025.1639267

**Published:** 2025-06-18

**Authors:** Amal Al-Khotani, Nikolaos Christidis, Essam Ahmed Al-Moraissi

**Affiliations:** ^1^ Dental Department, East Jeddah Hospital, Ministry of Health, Jeddah, Saudi Arabia; ^2^ Division of Oral Rehabilitation, Department of Dental Medicine, Karolinska Institutet, Huddinge, Sweden; ^3^ Department of Oral and Maxillofacial Surgery, Faculty of Dentistry, Thamar University, Thamar, Yemen

**Keywords:** pediatric dentistry, orthodontic, intraoral 3D scanning, general anesthesia, growth and development, social determinants

**Editorial on the Research Topic**
Uncovering a multidisciplinary approach in pediatric dentistry

Holistic dentistry is a general term that can have different interpretations among dental practitioners; it is defined as considering a patient's overall health, including both oral and general health (1). A holistic approach to pediatric dentistry involves closely monitoring a child's needs beyond conventional dental health, including general health, diet, sleep patterns, activity level, and lifestyle habits. This holistic approach emphasizes the interconnection between a child's oral health and their overall physical, emotional, and psychological well-being (2, 3). This interconnectedness addresses the need for more comprehensive approaches, which can be seen as the practical application of multidisciplinary principles in modern clinical practice. This editorial introduces the Research Topic, Uncovering a Multidisciplinary Approach in Pediatric Dentistry, in Frontiers in Dental Medicine. The present article collection was proposed in response to evolving demands in pediatric dentistry that call for a comprehensive, multidisciplinary approach that integrates clinical excellence, psychosocial care, and technological innovation. The contributions not only reflect ongoing improvements in the field but also highlight the beauty of interconnected research efforts.

This research topic includes eight articles published in this multidisciplinary context by 53 authors, reflecting the necessity of collaboration not only between medical and dental healthcare practitioners but also between different disciplines within the dental profession. Serrano-Velasco et al. conducted a systematic review that evaluated the perception, scanning time, and reliability of intraoral scanners for full-arch impressions in pediatric patients, aged 6–12 years. Of the studies that met the inclusion criteria, three analyzed patient perception and scanning or impression time, and two assessed the reliability and reproducibility of intraoral scanners. However, all studies reported that intraoral scanners were a more comfortable tool than the conventional method. Furthermore, the systematic review showed that although the reliability of digital impression technique is not clear, it is clinically acceptable. Moreover, Zhang et al. explored the relationship between atopic dermatitis (AD) and microbiota in children by comparing skin, oral, and gut microbiomes of 50 children with AD and 50 healthy controls using 16S rRNA sequencing. The results showed a significant decrease in oral microbiota diversity, together with changes in specific bacterial species in AD patients (S. mitis (40%), N. mucosa (7.3%), G. haemolysans (7.4%), and H. parainfluenzae (5.6%)). The changes in the oral microbiota composition in AD patients were found to differ from those in oral and chronic inflammatory diseases. These findings demonstrate the role of multi-site microbiota in AD pathogenesis and offer insights for future diagnostics and therapeutic strategies.

A study by Jiménez-Lobo et al. aimed to assess changes in the oral health-related quality of life of 80 Costa Rican schoolchildren aged 8–12, before and after dental treatment. This study found a high prevalence of dental caries (56.1%), hypomineralization (53.7%), and malocclusions (82.9%). After treatment, a slight reduction in the Simplified Oral Hygiene Index was noted, whereas its mean decreased from 53.7 ± 7.8 to 31.4 ± 4.2. Despite unchanged sociodemographic conditions, dental treatment significantly improved the children's perceived oral health and quality of life, highlighting the impact of clinical care regardless of oral health disparities. This improvement in quality of life may significantly affect children's social condition, including their relationships with family or neighbors. In this context, Lee et al. addressed the association between children's oral health and their neighborhoods as a social determinant. They examined how neighborhood context, as measured by the Area Deprivation Index (ADI), influences toothbrushing frequency and plaque levels in Medicaid-enrolled children in urban Chicago. The authors' analysis showed that the ADI was significantly associated with higher plaque scores, suggesting that neighborhoods have a significant disadvantage in oral hygiene quality rather than its frequency. This study concluded that socioeconomic and environmental factors considerably impact the design of equitable preventive healthcare initiatives.

Age-appropriate care is a cornerstone of pediatric dentistry. It integrates preventive and functional treatment strategies through multidisciplinary collaboration in order to address the unique needs of growing children (4). In this regard, two of the articles in our collection presented common orthodontic problems that occur early in life and therefore need early diagnosis by a pediatric dentist and early intervention from an orthodontist. One of them is the root resorption of the lateral permanent incisors. Helal et al. aimed to assess the effect of interceptive extraction of the primary canines on the condition of the roots of the adjacent permanent teeth to mesioangular displaced canines by comparing the extraction group to the non-extraction group. The authors found no significant difference in resorption levels between the two groups. They also reported that root resorption occurred in more than a quarter of the cases, with a substantial increase in severity at the one-year follow-up. However, the severity of the resorption was observed more in palatally positioned canines than in buccally or mid-alveolarly displaced canines. These findings indicate that timely clinical assessment is crucial for the early detection and possible intervention in cases of mesioangular canine displacement.

In a case series report, Maruya et al. showed their management of another orthodontic issue: severely inversely impacted maxillary incisors. In this study, the authors examined the orthodontic treatment for severely inversely impacted maxillary central incisors and presented them in a series of case studies. They successfully repositioned all incisors into their correct position during the early stage of incisor development before root formation progressed. This repositioning appears to be both safe and successful due to the collaborative work between the oral surgeon and the orthodontist. In this regard, Damanhouri et al. reported a case that shares some similarities in collaborative treatment between different dental specialties. A 10-year-old boy attended the pediatric dental clinic complaining about his ugly front teeth. He was bullied at school due to a supernumerary tooth fused to the right central incisor. This negatively affected his psychosocial well-being, as his mother informed the authors. Examination showed not only a fused anterior tooth but also supernumerary teeth underneath. Combined management was performed by a pediatric dentist, an oral and maxillofacial surgeon (OMFS), and a psychiatrist. The OMFS extracted the affected tooth with its adjacent tooth under general anesthesia (GA) to allow eruption of the supernumerary impacted teeth in place. This was due to the difficulty in removing the underlying supernumerary teeth near the nasal floor. The child's level of anxiety decreased, especially after the construction of an aesthetic removable appliance by the pediatric dentist.

It is worth noting that dental treatment under GA has become an essential intervention for many pediatric patients, particularly those with complex dental conditions, behavioral management difficulties, or certain medical conditions/disabilities (5). This necessity highlights the value of a multidisciplinary approach in pediatric dental care, where coordinated efforts between pediatric dentists, anesthesiologists, pediatricians, psychologists, and nursing staff ensure a safe and efficient treatment pathway. Al-Khotani et al. demonstrated that dental treatment under GA is one way to manage children's behavior, including those with special healthcare needs (SHCN). The authors compared the clinical profiles and treatment outcomes of healthy patients to those of SHCN patients who underwent dental rehabilitation under GA. Their study found that SHCN patients experience extended hospitalization and require more complex treatment plans than their healthy peers.

In conclusion, this Research Topic illustrates the dynamic potential of a multidisciplinary approach in advancing pediatric dentistry. The contributions demonstrate how collaboration across dental specialties, and equally important, with other healthcare professionals, enhances clinical outcomes and supports the holistic well-being of children. From technological innovations to social determinants of health, the studies collectively underscore the importance of individualized, age-appropriate, and context-aware care. By integrating psychosocial, medical, and dental perspectives, pediatric dentistry is poised to meet the evolving needs of today's diverse child populations. This editorial reinforces the value of shared knowledge and interdisciplinary synergy, paving the way for future innovations in pediatric oral healthcare.
